# Integrated 16S rRNA Sequencing and Metabolomic Analyses Reveal Gut Microbiota Dysbiosis and Metabolic Perturbations in Neonatal Dairy Calves with Bovine Rotavirus-Induced Diarrhea

**DOI:** 10.3390/biology15110855

**Published:** 2026-05-29

**Authors:** Youli Yu, Yuxi Zhao, Wei He, Zhengqing Yu, Yuqiu Yang, Jiandong Wang

**Affiliations:** 1 Institute of Animal Science, Ningxia Academy of Agricultural and Forestry Sciences, No. 590, Huanghe East Road, Jinfeng District, Yinchuan 750002, China; 2 Ningxia Hui Autonomous Region Food Testing and Research Institute, No. 577, Fengyue Lane, Zhengyuan South Street, Jinfeng District, Yinchuan 750002, China; 3 College of Animal Science and Technology, Ningxia University, No. 539, Helanshan West Road, Xixia District, Yinchuan 750021, China

**Keywords:** bovine rotavirus, diarrhea dairy calf, 16S rRNA sequencing, gut microbiota, metabolomics, multi-omics integration, microbial dysbiosis, lipid metabolism

## Abstract

Bovine rotavirus (BRV) is a major pathogen causing diarrhea in neonatal dairy calves, yet its impact on the gut microbiome and host metabolism remains incompletely understood. This study integrated 16S rRNA gene sequencing and untargeted metabolomics to characterize the fecal microbial and metabolic profiles of BRV-infected diarrheic calves compared to healthy controls. Fecal samples were collected from 16 Holstein calves (<30 days old), equally divided into a BRV-infected group (confirmed by RT-qPCR) and a healthy control group. Alpha diversity analysis revealed a significant reduction in microbial richness (Chao1, *p* = 0.012) in the BRV group. Beta diversity (Jaccard distance) showed distinct clustering between groups (PERMANOVA, *p* < 0.05). Linear discriminant analysis effect size (LEfSe) identified a significant enrichment of *Escherichia* (*Proteobacteria*) and *Enterococcus* (*Firmicutes*) in BRV calves, whereas healthy controls were enriched in *Blautia* and *Faecalibacterium* (both *Firmicutes*, with *Faecalibacterium* also commonly associated with healthy gut communities). Metabolomic analysis via UHPLC-MS/MS demonstrated a clear separation of metabolic profiles. BRV infection was associated with significant alterations in lipid metabolism pathways, notably elevated levels of lysophosphatidylcholines (LPCs) and lysophosphatidylethanolamines (LPEs). Integrated Spearman correlation analysis revealed extensive and significant associations (|ρ| > 0.6, FDR-adjusted *p* < 0.05) between differential bacterial genera (e.g., *Enterococcus*, *Escherichia*) and differential metabolites (e.g., glycerolipids, amino acid derivatives), suggesting a close linkage between gut microbial dysbiosis and metabolic remodeling during BRV infection. These findings provide a multi-omics perspective on the interplay between the gut microbiota and host metabolism in BRV-induced diarrhea, offering potential insights for developing microbiota-based diagnostic or therapeutic strategies.

## Simple Summary

Bovine rotavirus (BRV) is a common virus that causes severe diarrhea in newborn dairy calves, leading to significant health problems and economic losses for farmers. However, it is not fully understood how this virus affects the community of bacteria living in the calves’ guts (the gut microbiome) and the chemical processes (metabolites) produced by the body. In this study, we compared 8 calves with BRV infection and 8 healthy calves without BRV. We analyzed the bacteria in their feces and the small chemical molecules present. The results showed that infected calves had fewer types of gut bacteria and a different bacterial composition compared to healthy calves. Specifically, harmful bacteria like *Enterococcus* (*Proteobacteria*) and *Escherichia* (*Firmicutes*) increased, while beneficial bacteria such as *Blautia* and *Faecalibacterium* decreased. The infected calves also showed changes in many metabolites, especially those related to fat metabolism. Importantly, these bacterial changes were closely linked to the metabolic changes, suggesting that the virus disrupts the normal balance of gut microbes, which in turn alters the calf’s metabolism. These findings help us better understand how BRV causes diarrhea and may lead to new ways to diagnose or treat the disease by targeting the gut microbiome.

## 1. Introduction

Neonatal calf diarrhea (NCD) remains one of the most significant health challenges in dairy farming worldwide, accounting for more than 50% of calf mortality and contributing to substantial economic losses through increased morbidity, treatment costs, reduced milk production, and impaired long-term productivity [1,2]. Among the various etiological agents responsible for NCD, bovine rotavirus (BRV) stands out as a primary pathogen, particularly affecting calves during their first month of life. The pathogenesis of rotavirus-associated diarrhea extends beyond direct viral damage to intestinal epithelium, involving complex interactions between the pathogen, host immune system, and the gut microbiome.

Recent advances in high-throughput sequencing technologies have revolutionized our understanding of the gut microbiota’s role in health and disease. The 16S rRNA gene amplicon sequencing has emerged as a powerful and cost-effective tool for routine microbial identification, enabling researchers to characterize the complex interactions between host, pathogen, and microbiome communities [3]. Studies employing 16S rRNA sequencing have provided critical insights into the microbial ecology of calf diarrhea. For instance, a comprehensive study analyzing 40 fecal samples from diarrheic and healthy neonatal calves using full-length 16S rRNA sequencing revealed that diarrheic calves exhibited significantly reduced alpha diversity measures, including Chao1, Shannon, and Simpson indices, indicating marked dysbiosis [4]. Similarly, Slanzon et al. [5] demonstrated that fecal microbiome profiles varied significantly with the severity of gastrointestinal disease in neonatal dairy calves, with more severe cases showing greater microbial disruption.

The gut microbiota plays a crucial role in maintaining intestinal homeostasis, and disruptions in microbial community structure have been closely linked to gastrointestinal disorders. Multiple studies have consistently shown that BRV infection induces significant alterations in the gut microbiota composition of infected calves [6,7]. Specifically, rotavirus infection has been associated with decreased bacterial diversity and richness, characteristic features of gut dysbiosis [6,7]. At the phylum level, diarrheic calves demonstrate a notable increase in the relative abundance of Proteobacteria and Actinobacteriota, while showing a significant decrease in Bacteroidetes [8]. At the genus level, these changes include significant reductions in beneficial genera such as *Lactobacillus*, *Subdoligranulum*, *Blautia*, *Bifidobacterium*, *Bacteroides*, and *Faecalibacterium*, while pathogenic bacteria like *Escherichia-Shigella*, *Clostridium*, and *Fusobacterium* show increased abundance [9,10,11].

A large-scale longitudinal study tracking 57 calves from birth to post-weaning with 450 fecal samples revealed that successful fecal microbiota transplantation (FMT) increased the family Porphyromonadaceae and reduced fecal amino acid concentrations, which strongly correlated with diarrhea remission [12]. Furthermore, recent research has shown that rotavirus-infected calves exhibit bacterial dysbiosis characterized by lower diversity and fewer observed genera compared to healthy calves, with these alterations correlating with changes in physiological parameters such as white blood cell counts, blood urea nitrogen, serum amyloid protein A, and glucose concentrations [9]. The elevated abundance of Proteobacteria in rotavirus-infected calves reflects a state of dysbiosis that may exacerbate disease severity and compromise recovery [7].

While 16S rRNA sequencing provides valuable insights into microbial community composition, it offers limited information about functional capabilities and metabolic activities of the gut microbiome. To address this gap, metabolomics has emerged as a complementary approach to elucidate the biochemical consequences of microbiota alterations. Integrating microbiome and metabolome data has become essential for understanding host-microbe interactions and identifying predictive biomarkers for disease [13]. Recent multi-omics studies have demonstrated that BRV and bovine coronavirus infections alter not only fecal microbiota composition but also metabolite profiles significantly [10].

Shi et al. [2] performed comprehensive metagenomic and metabolomic analyses on 60 fecal samples from Xia-nan calves and found that diarrheic calves exhibited marked decreases in purines (adenosine, adenine, 2′-deoxyguanosine, allantoate, deoxyinosine, and deoxyguanosine) and arachidonic acid metabolites (prostaglandin F2α and prostaglandin E2) compared to healthy calves. These metabolic changes encompass multiple pathways, including disruptions in purine metabolism, arachidonic acid metabolism, phospholipid metabolism, and acid-base homeostasis [2,10]. further demonstrated that BRV and bovine coronavirus infections led to reduced levels of short-chain fatty acids (SCFAs) such as acetic acid and propionic acid in diarrheic calves, which was associated with the depletion of SCFA-producing bacteria, including Parabacteroides, Fournierella, and Collinsella. A seasonal dynamics study analyzing gut microbial–SCFA–immune crosstalk in diarrheic calves revealed that diarrheic calves had significantly higher levels of interleukin-1β, endotoxin, and diamine oxidase, and lower levels of interleukin-10 and immunoglobulins across all four seasons [14].

The integration of 16S rRNA sequencing with untargeted metabolomics represents a powerful multi-omics approach to comprehensively investigate the pathogenesis of rotavirus-associated diarrhea. This combined strategy enables researchers to identify specific microbiota–metabolite pairs that may serve as diagnostic markers or therapeutic targets. For instance, correlation analyses have revealed that certain bacterial taxa, such as Escherichia-Shigella, show significant positive correlations with inflammatory markers (endotoxin, diamine oxidase, IL-1β, and TNF-α) but negative correlations with IL-10 and IgA [15]. Similarly, Clostridium_sensu_stricto_1 was found to be positively correlated with endotoxin and diamine oxidase and negatively correlated with IL-10 and IgA [15]. Moreover, metabolites produced by gut microbiota, particularly short-chain fatty acids like butyrate, have demonstrated antiviral properties and immunomodulatory effects that may influence disease outcomes.

Recent studies have also explored therapeutic interventions based on microbiome modulation. Fan et al. [16] conducted a rational framework study for FMT efficacy in calf diarrhea treatment, achieving a 70% success rate across 20 treatment cases. Their machine learning analysis identified Selenomonas as showing significant donor–recipient compatibility in successful FMT treatments, and weighted gene correlation network analysis confirmed positively or negatively correlated pairs of bacterial taxa (family Veillonellaceae) and metabolomic features (amino acids and SCFAs) responsible for FMT success. A subsequent study comparing frozen and freeze-dried FMT formulations found that both treatments achieved 100% clinical effectiveness, with freeze-dried FMT showing superior performance in retaining donor microbiota in recipient calves [17]. Additionally, a study using machine learning-enhanced assessment identified nine Limosilactobacillus reuteri strains that effectively treated severe diarrhea in calves after antibiotic failure, with noticeable improvements in fecal morphology and animal behavior [15].

Despite these advances, several knowledge gaps remain regarding the precise mechanisms through which BRV infection disrupts the gut microbiota and how these microbial alterations contribute to metabolic dysfunction and disease severity. Most existing studies have focused on describing compositional changes in the microbiota or identifying differentially abundant metabolites, but few have comprehensively integrated both datasets to elucidate the functional relationships between specific microbial taxa and their metabolic outputs in the context of BRV infection. Understanding these microbiota–metabolite interactions is crucial for developing targeted interventions, such as probiotic therapies or nutritional strategies, to prevent and treat rotavirus-associated diarrhea in calves.

Therefore, this study aims to employ 16S rRNA gene sequencing and untargeted metabolomics to comprehensively characterize the alterations in gut microbiota composition and fecal metabolite profiles in calves with BRV-associated diarrhea. By integrating these multi-omics datasets, we seek to identify key microbial taxa and metabolic pathways that are disrupted during BRV infection, establish correlations between specific microbiota–metabolite pairs, and elucidate potential mechanisms underlying the pathogenesis of rotavirus-induced diarrhea. These findings will provide novel insights into the complex interplay between the gut microbiome, host metabolism, and viral infection, ultimately contributing to the development of more effective prevention and therapeutic strategies for neonatal calf diarrhea.

## 2. Materials and Methods

### 2.1. Ethical Statement

The animal study protocol was officially approved by the Animal Experiment Ethics Committee of the Ningxia Academy of Agricultural and Forestry Sciences (ethical approval code: NXNKYKJLL-2025-14, approval date: 17 March 2025).

### 2.2. Animals and Sample Collection

Fecal samples were collected from Holstein dairy calves aged < 30 days at Ningxia Wuzhong city from a commercial dairy farm. Calves were assigned to two groups based on clinical examination and laboratory confirmation: a bovine rotavirus (BRV)-infected diarrhea group (BRV group, *n* = 8) and a healthy control group (Cont group, *n* = 8). Diarrhea was defined using a fecal consistency scoring system, in which calves with fecal scores ≥ 2 (semi-liquid to watery) were classified as diarrheic [18]. All calves were female Holstein calves from the same commercial dairy farm, housed under identical management conditions, and fed the same colostrum and milk replacer regimen.

Fresh fecal samples were collected from the rectum of each calf using sterile gloves and immediately placed into sterile collection tubes. Samples were transported to the laboratory on ice within 2 h of collection and immediately stored at −80 °C until further analysis.

### 2.3. BRV Detection and Confirmation

#### 2.3.1. RNA Extraction

Total RNA was extracted from fecal samples using a commercial viral RNA extraction kit (RNeasy Mini Kit, Qiagen, Hilden, Germany) following the manufacturer’s instructions. Briefly, approximately 200 mg of fecal material was homogenized in lysis buffer, followed by centrifugation to remove debris. The supernatant was processed according to the kit protocol. RNA quality and quantity were assessed using a NanoDrop spectrophotometer (Thermo Fisher Scientific, Waltham, MA, USA), with A260/A280 ratios between 1.8 and 2.0 considered acceptable [19].

#### 2.3.2. RT-qPCR Detection of BRV

BRV group A was detected by reverse transcription quantitative real-time PCR (RT-qPCR) targeting the VP6 gene using previously validated primers and TaqMan probe [20].

RT-qPCR reactions were performed in a total volume of 20 μL containing 10 μL of 2× RT-qPCR master mix, 1 μL each of forward and reverse primers (10 μM), 1 μL of TaqMan probe (10 μM), 2 μL of RNA template, and 5 μL of nuclease-free water. The thermal cycling conditions consisted of reverse transcription at 50 °C for 15 min, initial denaturation at 95 °C for 2 min, followed by 40 cycles of denaturation at 95 °C for 15 s and annealing/extension at 60 °C for 1 min. All reactions were performed in duplicate on an ABI Step One Plus Real-Time PCR Detection System (Thermo Scientific, Waltham, MA, USA). Calves in the healthy control group were also tested by the same RT-qPCR assay and confirmed negative for BRV.

### 2.4. 16S rRNA Gene Sequencing

#### 2.4.1. DNA Extraction

Fecal microbial DNA was extracted from 200 mg of fecal samples using a commercial DNA extraction kit specifically designed for fecal material, E.Z.N.A. Stool DNA Kit (Omega Bio-tek, Norcross, GA, USA), following the manufacturer’s protocol.

#### 2.4.2. Library Preparation and Sequencing

The V3-V4 hypervariable region of the bacterial 16S rRNA gene was amplified using the universal primers 338F (5′-ACTCCTACGGGAGGCAGCA-3′) and 806R (5′-GGACTACHVGGGTWTCTAAT-3′) with barcode sequences added to the 5′ end of each primer for multiplexing [21]. PCR was performed in 25 μL reactions containing 12.5 μL 2× Taq PCR Master Mix (Tiangen, Beijing, China), 1 μL each primer (10 μM), 1 μL template DNA (10 ng/μL), and 9.5 μL nuclease-free water. Cycling conditions were: 95 °C for 5 min; 30 cycles of 95 °C for 30 s, 55 °C for 30 s, and 72 °C for 45 s; and 72 °C for 10 min.

Amplicons were verified by 2% agarose gel electrophoresis and purified by using AxyPrep DNA Gel Extraction Kit (Axygen, Union City, CA, USA). Purified products were quantified using a Qubit dsDNA HS Assay Kit (Thermo Fisher Scientific, Waltham, MA, USA), pooled in equimolar amounts, and used for library preparation with the Illumina TruSeq DNA PCR-Free Library Preparation Kit (Illumina, San Diego, CA, USA). Sequencing was performed on an Illumina NovaSeq 6000 platform (Illumina, San Diego, CA, USA) with paired-end reads (2 × 250 bp) [22].

#### 2.4.3. Sequence Data Processing and Analysis

Raw sequencing data were demultiplexed and quality-filtered using QIIME 2 (v 2019.1) [23]. Primer sequences were removed using the q2-cutadapt plugin [24]. Denoising, chimera removal, and paired-end merging were performed using the DADA2 plugin. with truncation parameters set according to quality profiles (--p-trunc-len-f 240 and --p-trunc-len-r 220 for 2 × 250 bp data) [25]. Amplicon sequence variants (ASVs) were generated after removal of chimeric sequences.

Taxonomic classification was performed using the q2-feature-classifier plugin with a pre-trained Naive Bayes classifier based on the Greengenes2 database [26], trimmed to the V3–V4 region.

Alpha diversity metrics, including observed features, Shannon diversity index, Simpson index, and Chao1 richness estimator, were calculated using the q2-diversity plugin after rarefying samples to an even sequencing depth determined based on rarefaction curves. Beta diversity was assessed using weighted and unweighted UniFrac distances, Bray–Curtis dissimilarity, and Jaccard distance [27]. Principal coordinates analysis (PCoA) was performed to visualize beta diversity patterns, and statistical significance was evaluated using permutational multivariate analysis of variance (PERMANOVA) with 999 permutations [28]. Linear discriminant analysis effect size (LEfSe) was used to identify differentially abundant bacterial taxa between groups with an LDA score threshold > 2.0 and *p* < 0.05 [29].

### 2.5. Untargeted Metabolomics Analysis

#### 2.5.1. Sample Preparation for Metabolomics

Fecal samples were lyophilized for 48 h to remove moisture and improve homogenization [30,31]. Approximately 50 mg of freeze-dried material was extracted with 1 mL pre-chilled methanol:water (4:1, *v*/*v*) containing internal standards (internal standard mix, Sigma-Aldrich, St. Louis, MI, USA) [32]. These internal standards were distinct from the reference standards used for metabolite identification and were added solely for data normalization and quality control purposes. Samples were bead-beaten (Precellys 24, Bertin Technologies, Montigny-le-Bretonneux, France) at 6500 rpm for 60 s, repeated three times with 30 s intervals on ice. The homogenate was vortexed (30 s), sonicated in an ice-water bath (10 min), incubated at −20 °C for 1 h, and centrifuged at 13,000× *g* for 15 min at 4 °C [33]. The supernatant was transferred and dried under nitrogen, reconstituted in 200 μL methanol:water (1:1, *v*/*v*), centrifuged (13,000× *g*, 10 min, 4 °C), and the final supernatant was transferred to LC–MS vials.

A pooled quality control (QC) sample was prepared by mixing equal aliquots of all extracts, and extraction blanks were included to monitor background signals [31].

#### 2.5.2. LC-MS Analysis

Untargeted metabolomics was performed using UHPLC coupled to a Q Exactive Orbitrap MS (Thermo Fisher Scientific, Waltham, MA, USA) [34]. Chromatographic separation was achieved using an ACQUITY UPLC HSS T3 column (2.1 × 100 mm, 1.8 μm particle size, Waters, Milford, MA, USA). The mobile phases consisted of (A) 0.1% formic acid in water and (B) 0.1% formic acid in acetonitrile (LC-MS grade, Fisher Scientific, Waltham, MA, USA). The gradient elution program was as follows: 0–1 min, 5% B; 1–9 min, 5–95% B; 9–12 min, 95% B; 12–12.1 min, 95–5% B; 12.1–15 min, 5% B for column re-equilibration. The flow rate was set at 0.4 mL/min, and the injection volume was 2 μL.

Data were acquired in positive and negative ESI modes over m/z 50–1200 in centroid mode. Lock-mass correction was applied using leucine enkephalin (m/z 556.2771 in positive mode and m/z 554.2615 in negative mode) [31]. QC injections were performed at regular intervals (every 10 samples). Features with a QC coefficient of variation (CV) > 30% were excluded.

#### 2.5.3. Metabolomics Data Processing

Raw LC-MS data files were converted to mzXML format using MSConvert (proteowizard 3.0.7069) [35]. Peak detection, alignment, and integration were performed using XCMS software (version 3.12.0) in the R environment [36]. The XCMS parameters were optimized for the specific chromatographic and mass spectrometric conditions used. Features detected in blank samples or with CV > 30% in QC samples were removed from the dataset.

Metabolite identification was performed using accurate mass matching (mass error < 10 ppm), retention time comparison with authentic standards when available, and MS/MS fragmentation pattern matching against metabolite databases including METLIN (https://metlin.scripps.edu, accessed 18 March 2026), HMDB (Human Metabolome Database, https://hmdb.ca, accessed 18 March 2026), and KEGG (Kyoto Encyclopedia of Genes and Genomes, https://www.genome.jp/kegg/, accessed 18 March 2026) [37,38]. Metabolites were assigned identification confidence levels according to the Metabolomics Standards Initiative guidelines [39].

### 2.6. Integrated Multi-Omics Data Analysis

#### 2.6.1. Statistical Analysis

Statistical analyses were performed using R software (version 4.3.2). For microbiome data, differences in alpha diversity metrics between groups were tested using the Kruskal–Wallis test followed by Dunn’s post hoc test with Benjamini–Hochberg false discovery rate (FDR) correction. Beta diversity differences were assessed using PERMANOVA with 999 permutations. For metabolomics data, features were log-transformed and auto-scaled prior to multivariate analysis. Unsupervised principal component analysis (PCA) and supervised partial least squares-discriminant analysis (PLS-DA) were performed using the mixOmics package in R (v 4.3.2) [40]. Model performance was evaluated using 10-fold cross-validation and permutation tests (*n* = 1000).

#### 2.6.2. Microbiome-Metabolome Correlation Analysis

To identify associations between gut microbiota composition and fecal metabolite profiles, Spearman’s rank correlation analysis was performed between the relative abundances of bacterial genera (present in >50% of samples with mean relative abundance > 0.1%) and the normalized intensities of identified metabolites. Correlations with |ρ| > 0.6 and FDR-adjusted *p* < 0.05 were considered significant.

## 3. Results

### 3.1. Alpha and Beta Diversity

**Figure 1:**
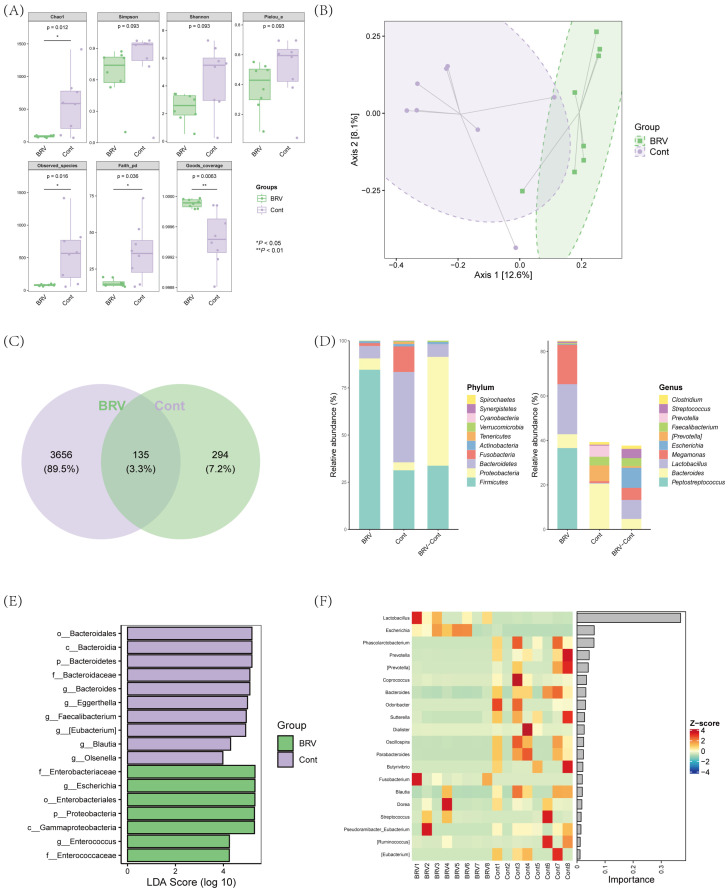
Alterations in gut microbiota diversity and composition following BRV infection. (**A**) Alpha diversity indices. Chao1 and observed species were significantly reduced, while Shannon, Simpson, and Pielou’s evenness showed decreasing trends (not significant). Good’s coverage was significantly higher in BRV group. *p* < 0.05. (**B**) PCoA based on Jaccard distance showing clear separation between groups. (**C**) Venn diagram of ASVs. 89.5% were shared, 7.2% unique to BRV, 3.3% unique to Cont. (**D**) Relative abundance at phylum level. Dominant phyla were Firmicutes, Bacteroidetes, and Proteobacteria. (**E**) Differentially abundant taxa identified by LEfSe (LDA > 2, *p* < 0.05). BRV group enriched in *Escherichia* (*Proteobacteria*) and *Enterococcus* (*Firmicutes*); control group enriched in *Blautia* and *Faecalibacterium* (*Firmicutes*). (**F**) Top phyla contributing to group separation by random forest analysis.

Alpha diversity analysis demonstrated a significant reduction in microbial richness in the BRV group compared with the control group. Both the Chao1 richness estimator and the observed species index were significantly lower in BRV samples (Chao1, *p* = 0.012; observed species, *p* = 0.016; Figure 1A). In contrast, no statistically significant differences were observed in Shannon diversity index, Simpson index, or Pielou’s evenness, although all three indices exhibited a decreasing trend in the BRV group (*p* = 0.093). Good’s coverage was significantly higher in the BRV group (*p* = 0.0063), indicating sufficient sequencing depth and reduced community complexity.

Beta diversity analysis based on Jaccard distance revealed a clear separation between the BRV and control groups in principal coordinates analysis (PCoA) (Figure 1B). The first two principal coordinates explained 12.6% and 8.1% of the total variation, respectively. BRV samples clustered more tightly, whereas control samples displayed greater inter-individual variability, suggesting a distinct alteration in microbial community structure following BRV infection. Statistical significance of group separation was evaluated using PERMANOVA with 999 permutations.

### 3.2. Shared and Group-Specific ASVs Between BRV and Control Groups

Venn diagram analysis at the ASV level showed that the majority of microbial features were shared between the BRV and control groups, with 3656 ASVs (89.5%) detected in both groups. In addition, 294 ASVs (7.2%) were uniquely observed in the BRV group, whereas 135 ASVs (3.3%) were specific to the control group. These results indicate that although a conserved core microbiota was maintained between groups, BRV infection was associated with a greater number of group-specific ASVs, suggesting selective alterations in microbial community composition rather than a complete loss of microbial taxa (Figure 1C).

### 3.3. Microbial Composition at the Phylum and Genus Levels

To further characterize the compositional differences in gut microbiota between groups, the relative abundance of bacterial taxa was analyzed at both the phylum and genus levels. At the phylum level, the microbial communities of both groups were dominated by Firmicutes, Bacteroidetes, and Proteobacteria, together accounting for the majority of total sequences (Figure 1D). Although these dominant phyla were shared between the BRV and control groups, their relative abundance patterns differed between groups. At the genus level, several predominant genera exhibited distinct abundance distributions between BRV and control samples, indicating compositional shifts in the gut microbiota associated with BRV infection.

### 3.4. Differentially Abundant Taxa Identified by LEfSe

To identify specific bacterial taxa contributing to the compositional differences between groups, LEfSe was performed with an LDA score threshold greater than 2.0 and *p* < 0.05. LEfSe analysis revealed multiple differentially abundant taxa across different taxonomic levels between the BRV and control groups (Figure 1E).

Taxa enriched in the BRV group were primarily affiliated with the phyla Proteobacteria and Firmicutes. Proteobacteria enrichment included *Gammaproteobacteria*, *Enterobacteriales*, and the *Enterobacteriaceae* family (e.g., *Escherichia*), while *Firmicutes* enrichment was represented by *Enterococcus*. At the genus level, both *Escherichia* and *Enterococcus* were identified as key discriminative taxa associated with the BRV group.

In contrast, the control group was characterized by a higher abundance of taxa belonging to the phylum Bacteroidetes, particularly Bacteroidia and Bacteroidales. Several genera commonly associated with a complex gut microbial ecosystem, including Blautia and Faecalibacterium, were significantly enriched in control samples. The differential bacterial taxa between the BRV and control groups were identified using LEfSe analysis, with the detailed results provided in Table S1.

### 3.5. Key Microbial Taxa Identified by Random Forest Analysis

A random forest classification model was applied to identify key microbial taxa contributing to the discrimination between the BRV and control groups. Feature importance ranking based on the random forest model revealed that taxa affiliated with *Bacteroidetes*, *Proteobacteria*, and *Firmicutes* were the most influential contributors to group separation at the phylum level (Figure 1F).

### 3.6. Metabolic Profile Overview and Data Quality Assessment

To obtain an overview of the metabolic profiles, non-targeted metabolomic analysis was performed on samples from the BRV and control groups. After data preprocessing and normalization, high-quality metabolic features were retained for downstream multivariate and univariate analyses. No obvious outliers were observed, indicating overall data stability and suitability for subsequent statistical analysis.

### 3.7. Multivariate Analysis of Metabolic Profiles

**Figure 2:**
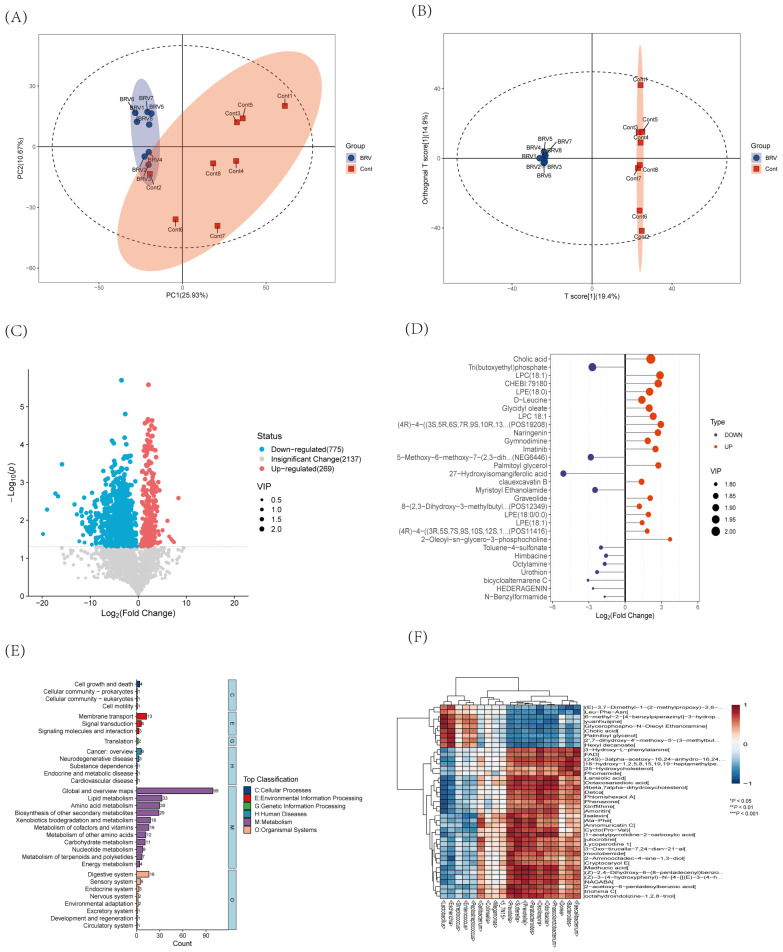
Metabolic profile alterations and microbiota–metabolite correlations after BRV infection. (**A**) PCA score plot. PC1 and PC2 explained 25.93% and 10.67% of variance. BRV samples clustered more tightly. (**B**) OPLS-DA score plot showing clear separation between BRV and control groups. (**C**) Differential metabolites (VIP > 1, FDR < 0.05, |log_2_FC| > 1). 269 metabolites increased and 775 decreased in the BRV group. (**D**) Representative differential metabolites. Lipid-related metabolites (LPCs, LPEs, glycerolipid derivatives) were elevated, while multiple small-molecule metabolites decreased in the BRV group. (**E**) KEGG pathway classification of differential metabolites, predominantly enriched in lipid, amino acid, carbohydrate, and energy metabolism. (**F**) Spearman correlation between differential bacterial genera and top 41 differential metabolites (*p* < 0.05). *Enterococcus*, *Escherichia*, *Prevotella*, *Faecalibacterium*, and *Lactobacillus* showed extensive correlations.

To explore the global metabolic differences between the BRV and control groups, a combination of unsupervised and supervised multivariate analyses was performed. Principal component analysis (PCA) revealed an overall separation tendency between the two groups, with PC1 and PC2 explaining 25.93% and 10.67% of the total variance, respectively (Figure 2A). Notably, samples from the BRV group clustered more tightly, whereas control samples showed greater dispersion, indicating distinct metabolic profiles between groups.

To further enhance group discrimination and assess metabolic patterns associated with BRV infection, orthogonal partial least squares discriminant analysis (OPLS-DA) was subsequently conducted. OPLS-DA score plots demonstrated a clear separation between the BRV and control groups (Figure 2B), confirming that BRV infection was a major contributor to the observed metabolic variation. Collectively, these multivariate analyses consistently demonstrate pronounced metabolic differences between the two groups.

### 3.8. Identification of Differential Metabolites Between BRV-Infected and Control Groups

Applying the thresholds of VIP > 1, FDR < 0.05, and |log_2_FC| > 1, we delineated a total of 1044 metabolites whose abundance differed significantly between the BRV and Count groups, comprising 269 elevated and 775 reduced species in the BRV cohort (Figure 2C).

A set of metabolites exhibited significant alterations following BRV infection. Notably, several lipid-related metabolites, including lysophosphatidylcholines (LPCs), lysophosphatidylethanolamines (LPEs), and glycerolipid derivatives, were significantly increased in the BRV-infected group, whereas multiple small-molecule metabolites were decreased (Figure 2D).

### 3.9. Functional Classification and Pathway Analysis of Differential Metabolites

To gain insight into the functional implications of metabolic alterations associated with BRV infection, differential metabolites were annotated using the KEGG database. KEGG pathway classification revealed that the differential metabolites were predominantly enriched in metabolism-related pathways, particularly lipid metabolism, amino acid metabolism, carbohydrate metabolism, and energy metabolism. In addition, pathways related to metabolism of cofactors and vitamins, xenobiotic biodegradation and metabolism, and biosynthesis of other secondary metabolites were also represented, indicating extensive metabolic remodeling associated with BRV infection (Figure 2E).

### 3.10. Integrated Correlation Analysis of Gut Microbiota and Metabolomic Profiles

To further explore the relationships between gut microbial alterations and metabolic changes associated with BRV infection, a Spearman correlation analysis was performed between differentially abundant bacterial genera and the top 41 differential metabolites. This analysis identified a substantial number of significant associations (*p* < 0.05), indicating extensive coordinated variation between microbial and metabolic features (Figure 2F). The full correlation results are provided in Table S2, and the abbreviations of all metabolites are listed in Table S3.

At the global level, multiple bacterial genera exhibiting differential abundance between groups showed significant correlations with a broad spectrum of metabolites, suggesting that metabolic alterations associated with BRV infection are closely linked to changes in gut microbial composition rather than isolated metabolic events.

Notably, the observed associations were not randomly distributed but displayed clear functional patterns. A large proportion of significant correlations involved lipid-related metabolites, including glycerolipids and lysophospholipids, as well as amino acid derivatives. These metabolites formed dense association clusters with several key bacterial genera, indicating potential modular relationships between microbial taxa and metabolic pathways.

Among the bacterial genera showing extensive correlations, Enterococcus, *Escherichia*, *Prevotella, Faecalibacterium*, and *Lactobacillus* were associated with multiple metabolites, highlighting their central positions within the microbiota–metabolite correlation network. Conversely, certain metabolites were correlated with multiple bacterial genera, suggesting shared metabolic associations across different microbial taxa.

Collectively, these results demonstrate that alterations in gut microbiota composition are accompanied by coordinated metabolic changes, supporting a close linkage between microbial dysbiosis and metabolic remodeling in the context of BRV infection.

## 4. Discussion

BRV is one of the most significant etiological agents of neonatal calf diarrhea, causing substantial economic losses in dairy production worldwide. Despite considerable advances in understanding the viral pathogenesis, the complex interactions between BRV infection, gut microbiota dysbiosis, and metabolic alterations remain incompletely understood. In this study, we employed an integrated approach combining 16S rRNA gene sequencing and non-targeted metabolomics to comprehensively characterize the gut microbiota composition and metabolic profile changes associated with BRV infection in calves. Our findings provide novel insights into the microbiota–metabolite interactions that underlie the pathophysiology of rotavirus-induced diarrhea.

### 4.1. BRV Infection Induces Significant Gut Microbiota Dysbiosis

Our alpha diversity analysis demonstrated that BRV infection was associated with a significant reduction in microbial richness and diversity, as evidenced by decreased Chao1 richness estimator and observed species indices. This finding is consistent with previous studies in rotavirus-infected calves, which have consistently reported reduced bacterial diversity as a hallmark of dysbiosis. For instance, a recent study by Kim et al. observed significant decreases in both Shannon’s index and Bray–Curtis dissimilarity in rotavirus-infected Holstein calves, confirming dysbiosis as a characteristic feature of rotavirus infection [6]. Similarly, Murtaza et al. reported that rotavirus infection in Sahiwal calves resulted in diminished microbial diversity, with Proteobacteria showing higher abundance in infected animals [7].

The reduced microbial diversity observed in our study may be attributed to multiple mechanisms. First, rotavirus-induced intestinal epithelial damage and inflammation create a hostile environment that selectively favors certain bacterial taxa while suppressing others. Second, rotavirus infection disrupts intestinal homeostasis through alterations in pH, oxygen availability, and nutrient gradients, which collectively reshape the microbial community structure. Third, the diarrhea itself may contribute to microbial washout, leading to the loss of commensal bacteria. These mechanisms are supported by recent evidence demonstrating that gut microbiota plays a critical role in defending against viral infections, and that dysbiosis can increase susceptibility to pathogen colonization [41].

The beta diversity analysis revealed distinct separation between the BRV-infected and control groups, with infected samples clustering more tightly than control samples. This suggests that rotavirus infection drives a convergent shift in microbial community structure, potentially reflecting common host responses to viral infection. The greater inter-individual variability observed in control samples may represent the natural diversity of healthy gut microbiota, which becomes constrained during infection toward a more uniform dysbiotic state.

### 4.2. Taxonomic Shifts: Enrichment of Opportunistic Pathogens and Depletion of Beneficial Commensals

LEfSe analysis identified distinct taxonomic signatures associated with BRV infection. Most notably, the BRV group exhibited significant enrichment of Proteobacteria, particularly members of the Enterobacteriaceae family, including Escherichia and Enterococcus. These findings align closely with previous reports in both human and animal models of rotavirus infection. Zhao et al. demonstrated that rotavirus-infected suckling mice showed enrichment of *Enterococcus* and *Escherichia*/*Shigella* genera, while beneficial genera such as Lactobacillus and Fusobacterium were depleted [42]. Similarly, in neonatal calves, rotavirus infection has been associated with increased abundance of *Escherichia*, *Clostridium*, and *Streptococcus* [43].

The expansion of Proteobacteria, particularly Enterobacteriaceae, is particularly concerning as this phylum is often considered a microbial signature of dysbiosis and epithelial dysfunction [44]. Proteobacteria are typically present in low abundance in healthy gut microbiota but can bloom during inflammatory conditions and epithelial damage [45]. The increase in facultative anaerobes such as *Escherichia* may be facilitated by rotavirus-induced epithelial disruption, which increases oxygen availability in the intestinal lumen and creates a favorable niche for these organisms [46,47]. Moreover, members of Enterobacteriaceae can exacerbate intestinal inflammation through lipopolysaccharide production and may contribute to prolonged diarrhea severity [48].

In contrast, the control group was characterized by higher abundances of *Bacteroidetes*, particularly *Bacteroidia* and *Bacteroidales*, as well as beneficial genera including *Blautia* and *Faecalibacterium*. These genera are well-recognized for their contributions to gut health through multiple mechanisms. *Faecalibacterium prausnitzii*, one of the most abundant bacteria in the healthy human and animal gut, is a major butyrate producer and has potent anti-inflammatory properties [49]. Blautia species have been associated with metabolic health and the production of short-chain fatty acids that support intestinal barrier integrity [50]. The depletion of these beneficial commensals during BRV infection may impair protective mechanisms, including barrier function, anti-inflammatory signaling, and colonization resistance against pathogens.

The loss of Bacteroidetes is particularly significant given their role in complex carbohydrate degradation and maintenance of intestinal homeostasis [51]. Bacteroidetes produce numerous polysaccharide-degrading enzymes and contribute to the production of acetate and propionate, which serve as energy sources for colonocytes and help maintain intestinal pH [52,53]. The reduction in these taxa during BRV infection may therefore compromise both nutritional status and intestinal barrier function, potentially prolonging recovery from diarrhea.

### 4.3. Metabolic Remodeling Associated with BRV Infection

Our metabolomic analysis revealed profound alterations in the metabolic landscape of BRV-infected calves, with significant changes spanning multiple biochemical pathways. The multivariate analyses (PCA and OPLS-DA) consistently demonstrated clear separation between BRV-infected and control groups, indicating that viral infection triggers extensive metabolic remodeling. Notably, BRV-infected samples exhibited tighter clustering compared to controls, suggesting convergent metabolic responses to infection that parallel the microbial community shifts observed in our microbiome analysis.

One of the most striking findings was the significant increase in lysophosphatidylcholines (LPCs) and lysophosphatidylethanolamines (LPEs) in BRV-infected animals. LPCs are bioactive lipid mediators derived from phosphatidylcholine through the action of phospholipase A2 and are increasingly recognized as important signaling molecules in inflammation and cellular stress. Elevated LPC levels have been reported in various pathological conditions, including cardiovascular disease and inflammatory disorders [54]. In the context of viral infection, increased LPC production may reflect several processes, including membrane remodeling, inflammatory responses, and oxidative stress.

The elevation of lysophospholipids in our study aligns with previous findings demonstrating the critical role of lipid metabolism in rotavirus replication. Multiple studies have shown that rotavirus infection induces lipid droplet biogenesis and that viral replication is highly dependent on cellular lipid metabolism. Criglar et al. demonstrated that rotavirus nonstructural protein NSP2 interacts with lipid droplet-associated proteins to facilitate viroplasm formation, the sites of viral genome replication and particle assembly [55]. Furthermore, Cisneros-Sarabia et al. showed that disruption of lipogenesis–lipolysis balance dissociates endoplasmic reticulum membranes from viroplasms and reduces infectious virus production [56].

The increased abundance of glycerolipid derivatives observed in our study is consistent with extensive lipid remodeling during rotavirus infection. Cheung et al. performed a comprehensive lipidomic analysis of rotavirus-infected cells and found that virtually all lipid classes were elevated during infection, with fatty acids showing the most dramatic increases [57]. The authors proposed that, as a consequence of high energy demands during viral replication, triacylglycerols are metabolized to fatty acids, which can then be utilized for membrane synthesis and energy production. This mechanism may explain the altered glycerolipid profile observed in our metabolomic analysis.

Our pathway enrichment analysis revealed that differential metabolites were predominantly associated with lipid metabolism, amino acid metabolism, carbohydrate metabolism, and energy metabolism. This finding is consistent with recent metabolomic studies of rotavirus infection in other models. Saha et al. demonstrated that rotavirus infection rewires host cellular metabolic pathways toward glutamine catabolism, with significant alterations in nucleotide biosynthesis, alanine/aspartate/glutamate metabolism, and the citric acid cycle [58]. Similarly, a metabolomic study of rotavirus-infected children identified galactose metabolism and butanoate metabolism as key pathways altered during infection, with butyrate emerging as a signature metabolic molecule [54].

The depletion of certain small-molecule metabolites in BRV-infected animals likely reflects disrupted intestinal absorption, altered microbial metabolism, and increased metabolic demands of the host immune response. Rotavirus infection damages the villous epithelium of the small intestine, leading to decreased absorption of nutrients, including sodium, glucose, and water, as well as reduced activity of brush border enzymes such as lactase, alkaline phosphatase, and sucrase [59,60]. These functional impairments would be expected to alter the metabolic profile of both intestinal contents and systemic circulation [61]. Furthermore, a recent comprehensive review systematically summarized the significance of cellular lipid metabolism for the replication of rotaviruses and other RNA viruses, highlighting that the interaction between rotavirus viroplasms and cellular lipid droplets is essential for infectious progeny virus production [62].

### 4.4. Integrated Microbiota–Metabolite Interactions

The correlation analysis between gut microbiota and metabolites revealed extensive coordinated variation, indicating that metabolic alterations associated with BRV infection are closely linked to changes in microbial community composition. It should be noted that the observed associations are correlative and do not imply direct causal relationships between specific microbial taxa and metabolites. Notably, multiple bacterial genera showing differential abundance between groups exhibited significant correlations with a broad spectrum of metabolites, particularly lipid-related compounds and amino acid derivatives. This finding underscores the integrated nature of host–microbe–metabolite interactions during viral infection.

Among the bacterial genera showing extensive metabolite correlations, *Enterococcus*, *Escherichia*, *Prevotella*, *Faecalibacterium*, and *Lactobacillus* emerged as central nodes in the microbiota–metabolite network. The enrichment of *Enterococcus* and *Escherichia* in BRV-infected animals, coupled with their strong correlations with altered metabolites, is consistent with a potential contribution of these organisms to the metabolic dysregulation observed during infection. Conversely, the depletion of beneficial genera such as *Faecalibacterium* and their association with depleted metabolites may reflect the loss of protective metabolic functions.

The dense association clusters observed between key bacterial genera and lipid metabolites are intriguing and may reflect several underlying mechanisms. First, certain gut bacteria can directly metabolize lipids and phospholipids, contributing to the lipid metabolite pool. Second, bacterial metabolites such as short-chain fatty acids can influence host lipid metabolism through effects on gene expression and metabolic enzyme activity. Third, inflammation induced by dysbiotic microbiota can activate phospholipases and alter membrane lipid composition, leading to increased production of lysophospholipids and other bioactive lipid mediators.

The observed microbiota–metabolite associations also suggest potential modular relationships between specific microbial taxa and metabolic pathways. For instance, the depletion of butyrate-producing bacteria such as *Faecalibacterium* and *Blautia* would be expected to reduce butyrate availability, which could compromise intestinal barrier function and anti-inflammatory signaling. Indeed, butyrate has been identified as a key metabolite altered during rotavirus infection and has demonstrated antiviral properties in experimental models [63]. The loss of butyrate production may therefore represent a critical mechanism linking microbiota dysbiosis to prolonged disease severity and delayed recovery.

### 4.5. Implications for Disease Pathogenesis and Therapeutic Interventions

Our findings have important implications for understanding the pathogenesis of rotavirus-induced diarrhea. The integrated analysis reveals that BRV infection triggers a cascade of events involving direct viral damage to enterocytes, disruption of gut microbiota homeostasis, and extensive metabolic remodeling. These changes likely contribute synergistically to disease severity and duration. The enrichment of opportunistic pathogens and depletion of beneficial commensals may perpetuate intestinal inflammation and barrier dysfunction beyond the acute phase of viral infection. Similarly, the alterations in lipid metabolism may both facilitate viral replication and compromise cellular energy homeostasis.

From a therapeutic perspective, our results suggest that interventions targeting the gut microbiota and metabolism may represent potential avenues to complement traditional supportive care for rotavirus-induced diarrhea. Probiotic supplementation with beneficial bacteria such as *Lactobacillus*, *Faecalibacterium*, or *Blautia* species could be explored in future studies to determine if they help restore microbial balance and accelerate recovery. Indeed, several studies have demonstrated that probiotic administration can reduce the duration of rotavirus diarrhea by approximately 1–2 days. The mechanisms likely involve competitive exclusion of pathogens, production of antimicrobial compounds, modulation of immune responses, and restoration of barrier function.

Nutritional interventions targeting specific metabolic pathways may also hold promise and warrant further investigation. For example, supplementation with short-chain fatty acids, particularly butyrate, could help restore barrier function and reduce inflammation. Similarly, interventions modulating lipid metabolism, such as fatty acid synthesis inhibitors, have shown potential as antiviral strategies in experimental models, although their clinical applicability requires further investigation. Prebiotics that selectively promote the growth of beneficial bacteria could represent another approach to restore microbiota homeostasis and protective metabolite production.

### 4.6. Limitations and Future Directions

Several limitations of this study should be acknowledged. First, the sample size (*n* = 8 per group) is relatively small for a multi-omics study. Although the results are statistically significant, larger cohort studies are warranted to validate these findings. Second, we did not screen the calves for other common enteric pathogens such as bovine coronavirus, *Cryptosporidium* spp., or enterotoxigenic *E. coli*; therefore, we cannot completely rule out the potential influence of undetected co-infections on the observed microbial dysbiosis and clinical signs. Third, our cross-sectional design provides a snapshot of microbiota and metabolic changes during infection but does not capture the dynamic temporal progression of these alterations. Longitudinal studies tracking changes from pre-infection through acute disease and recovery would provide valuable insights into causality and the sequence of events. Fourth, while our correlation analysis revealed associations between microbiota and metabolites, experimental validation through mechanistic studies is needed to establish causal relationships. Fifth, our study focused on fecal samples, which may not fully reflect changes occurring in different regions of the gastrointestinal tract.

Future research should aim to address these limitations through several approaches. Longitudinal sampling studies beginning before infection and continuing through recovery would clarify the temporal dynamics of microbiota–metabolite interactions. Metagenomic sequencing could provide functional insights into the metabolic capabilities of the altered microbiota. Targeted metabolomics focusing on specific pathways of interest, such as short-chain fatty acid production and lipid metabolism, could provide more detailed mechanistic information. Integration with transcriptomic and proteomic data would further enhance our understanding of host–microbe–metabolite interactions.

Additionally, interventional studies evaluating the efficacy of microbiota-targeted therapies, such as specific probiotic strains or fecal microbiota transplantation, would be valuable. Such studies should incorporate multi-omics approaches to understand how interventions affect the microbiota–metabolite network and whether restoration of these systems correlates with clinical improvement. Finally, comparative studies across different rotavirus strains and host species could reveal conserved versus strain-specific or host-specific mechanisms of microbiota–metabolite dysregulation.

## 5. Conclusions

In conclusion, this integrated 16S rRNA gene sequencing and metabolomics study provides comprehensive evidence that BRV infection in calves induces profound alterations in both gut microbiota composition and metabolic profiles. The infection is characterized by reduced microbial diversity, enrichment of opportunistic pathogens such as *Escherichia* and *Enterococcus*, depletion of beneficial commensals including *Faecalibacterium* and *Blautia*, and extensive metabolic remodeling, particularly affecting lipid metabolism. The strong correlations between microbiota and metabolites indicate that these changes are intimately connected and likely contribute synergistically to disease pathogenesis. These findings enhance our understanding of rotavirus-induced diarrhea and suggest that microbiota- and metabolism-targeted interventions may represent promising complementary therapeutic strategies. Further research is warranted to validate these findings, elucidate causal mechanisms, and translate these insights into clinical practice for improved management of rotavirus-associated disease in calves.

## Data Availability

The datasets supporting the conclusions of this article are included within the article and its Supplementary Files.

## Supplementary Materials

The following supporting information can be downloaded at: https://www.mdpi.com/article/10.3390/biology15110855/s1. Table S1: LEfSe-identified differential bacterial taxa between BRV and Cont groups; Table S2: Correlation analysis between microbes and metabolites; Table S3: Abbreviations and full names of metabolites used in this study.
